# The *Salmonella* Typhimurium Effector SpvB Subverts Host Membrane Trafficking by Targeting Clathrin and AP-1

**DOI:** 10.1016/j.mcpro.2023.100674

**Published:** 2023-11-02

**Authors:** Yi Yuan, Xinghao Wang, Jie Jin, Zhiheng Tang, Wei Xian, Xinyi Zhang, Jiaqi Fu, Kangmin He, Xiaoyun Liu

**Affiliations:** 1 Department of Microbiology and Infectious Disease Center, School of Basic Medical Sciences, Peking University Health Science Center, Beijing, China; 2 NHC Key Laboratory of Medical Immunology, Peking University, Beijing, China; 3 State Key Laboratory of Molecular Developmental Biology, Institute of Genetics and Developmental Biology, Chinese Academy of Sciences, Beijing, China; 4 University of Chinese Academy of Sciences, Beijing, China; 5 Department of Respiratory Medicine, Infectious Diseases and Pathogen Biology Center, Key Laboratory of Organ Regeneration and Transplantation of the Ministry of Education, State Key Laboratory for Zoonotic Diseases, The First Hospital of Jilin University, Changchun, China

**Keywords:** *Salmonella* Typhimurium, SpvB, clathrin, AP-1, membrane trafficking

## Abstract

*Salmonella enterica*, the etiological agent of gastrointestinal and systemic diseases, translocates a plethora of virulence factors through its type III secretion systems to host cells during infection. Among them, SpvB has been reported to harbor an ADP-ribosyltransferase domain in its C terminus, which destabilizes host cytoskeleton by modifying actin. However, whether this effector targets other host factors as well as the function of its N terminus still remains to be determined. Here, we found that SpvB targets clathrin and its adaptor AP-1 (adaptor protein 1) *via* interactions with its N-terminal domain. Notably, our data suggest that SpvB–clathrin/AP-1 associations disrupt clathrin-mediated endocytosis and protein secretion pathway as well. In addition, knocking down of AP-1 promotes *Salmonella* intracellular survival and proliferation in host cells.

## Highlights

• Protein interactome analysis identified host targets of *Salmonella* effector SpvB.
• SpvB targets clathrin and its adaptor AP-1 *via* interactions with its N-terminal domain.
• SpvB–clathrin/AP-1 interactions disrupt clathrin-mediated membrane trafficking.
• Knockdown of host AP-1 promotes intracellular *Salmonella* proliferation.

## Graphical abstract

**Figure:**
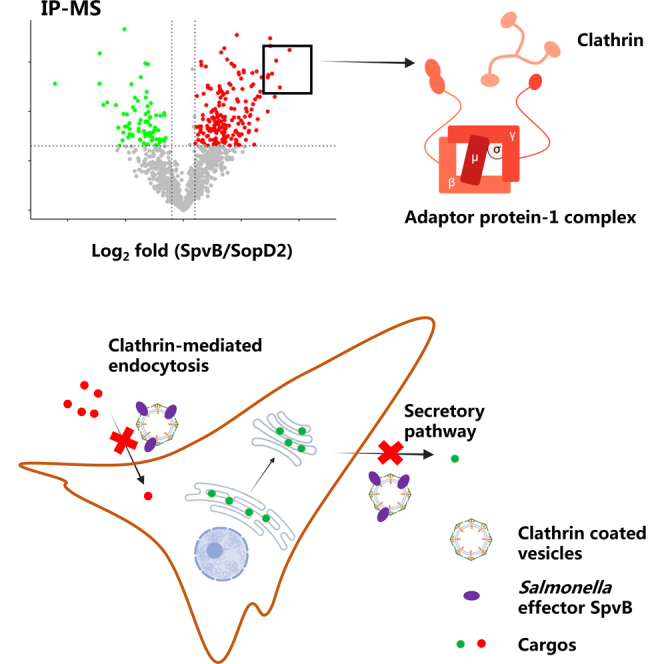

## In Brief

The human pathogen *Salmonella* inflicts millions of infections annually, and how this bacterium infects host cells remains poorly defined. Herein, we identified novel host substrates of its virulence factor SpvB *via* interactome analysis. SpvB inhibits host membrane trafficking processes by targeting a critical protein that coats transport vesicles known as clathrin as well as its adaptor protein AP-1 complex. These new findings of host–pathogen interactions may provide the basis for developing novel anti-infection strategies.

As a Gram-negative intracellular bacterial pathogen, *Salmonella enterica* causes acute gastroenteritis and more than 200,000 deaths worldwide per year (1). *S. enterica* serovar Typhimurium (referred as *S*. Typhimurium thereafter) causes nontyphoidal salmonellosis in a broad range of mammalian hosts including humans (2, 3). *S*. Typhimurium encodes two functionally distinct type III secretion systems (T3SSs) on *Salmonella* pathogenicity islands 1 and 2 (SPI-1 and SPI-2). These two T3SSs can inject virulence factors (also termed as effectors) into host cells and modulate diverse host cellular pathways (4). *Salmonella* plasmid virulence (*spv*) locus is a highly conserved 8-kb region in the pSLT virulence plasmid, which contains five members, *spvRABCD* (5). SpvB, SpvC, and SpvD are SPI-2 effectors, and their expression is transcriptionally regulated by SpvR and SpvA depending on growth conditions (6, 7, 8, 9, 10). SpvC is a phosphothreonine lyase that downregulates cytokine release during infection by targeting phosphorylated Erk (6). SpvD suppresses proinflammatory immune responses, and its structure reveals that it may be a cysteine protease (8, 11).

SpvB has two functional domains linked by a string of seven proline residues (nine in some serotypes) (12). Till now, most studies focus on its C terminus that alters host cytoskeleton by targeting Arg177 of actin for ADP-ribosylation (13, 14, 15). The N-terminal domain of SpvB exhibits significant sequence similarity to a secreted multicomponent insecticidal toxin TcaC from *Photorhabdus luminescens*, but its physiological function still remains unclear (7). Recent studies have shown versatile roles of SpvB in inhibition of NF-κB activity, disruption of intestinal epithelial barrier integrity, and dysregulation of iron metabolism during *Salmonella* infection (16, 17, 18). However, no additional cellular targets of SpvB, especially its N terminus, have been identified.

Clathrin is a cytosolic triskelion shape complex formed by trimerization of clathrin heavy and light chain subunits (19). Polymerization of these subunits leads to the formation of clathrin-coated vesicles that exert the function of cargo transport (20, 21, 22). However, clathrin does not bind to cargos directly. Instead, signal-dependent sorting of transmembrane cargos and packaging into vesicles are largely executed by clathrin adaptor protein (AP) complexes (20, 23). In this study, we found that the *Salmonella* SPI-2 T3SS effector SpvB interacts with clathrin and one of its adaptors AP-1, leading to inhibition of host endocytosis and protein secretion pathway. Therefore, our results illustrate a virulence mechanism of *S*. Typhimurium to hijack host membrane trafficking to promote pathogen infection.

## Experimental Procedures

### Plasmids

DNA for *spvB*, *sopF*, and *sopD2* was amplified from *S.* Typhimurium strain SL1344. Complementary DNA (cDNA) for human *AP1B*, *AP1G*, *AP1M*, *AP1S*, *CLTC*, and *ACTB* was amplified from reverse-transcribed cDNA of human embryonic kidney 293T (HEK293T) cells. The DNA was cloned into pcDNA4-FLAG-hemagglutinin (HA), pCS2-3×HA, or pCS2-enhanced GFP (EGFP) vectors for transient expression in mammalian cells.

### Bacteria and Cell Lines

The *S.* Typhimurium strain SL1344 and *Escherichia coli* strain DH5α were used in this study. The bacteria were routinely cultivated at 37 °C on LB plates with 1.5% agar or in LB medium with shaking. When necessary, antibiotics were added at the following concentrations: streptomycin (30 μg/ml) and ampicillin (100 μg/ml).

HEK293T and HeLa cells were obtained from American Type Culture Collection and cultured at 37 °C and 5% CO_2_ in Dulbecco's modified Eagle's medium (DMEM) (Gibco) supplemented with 10% fetal bovine serum. The genome-edited SUM159 cells were cultured at 37 °C and 5% CO_2_ in DMEM/F-12 with 20 mM Hepes (Corning) supplemented with 5% fetal bovine serum, 100 μg/ml penicillin and streptomycin, 5 μg/ml insulin, and 1 μg/ml hydrocortisone.

### Experimental Design and Statistical Rationale

Immunoprecipitation–mass spectrometry (IP–MS) experiments were employed to identify potential SpvB-interacting proteins. The *S*. Typhimurium T3SS effector SopD2 was used as a control. In total, we measured four biological replicates of paired SpvB and SopD2 IP samples in 18 LC–MS/MS experiments (for each sample, gel-fractionated proteins were processed and digested into two or three fractions). Label-free quantification was performed, and the proteins with the most significant differences were further selected for subsequent validation and functional experiments. All experiments were performed at least three times as independent replicates. Statistical analyses were conducted by using either Student's *t* test or one-way/two-way ANOVA followed by Tukey’s multiple comparison test within GraphPad Prism 7 (GraphPad Software, Inc), and *p* < 0.05 was considered as statistically significant.

### Transfection and IP

Transient transfection was performed using Lipofectamine 3000 (Invitrogen) or polyethyleneimine (PolySciences) following the manufacturers’ instructions.

For the identification of SpvB-interacting proteins, HEK293T cells (grown on a 10 cm dish) were transfected with FLAG-tagged SpvB or SopD2. In other cases, HEK293T cells from 6-well plates expressing proteins of interest were used. After 24 h of transfection, cells were washed with ice-cold PBS three times. Cells were lysed in lysis buffer containing 50 mM Tris–HCl (pH 7.5), 150 mM NaCl, and 1% Triton X-100 for 5 min at 4 °C. Soluble fractions were obtained by centrifuging at 13,000*g* for 10 min at 4 °C. About 40 μl of anti-FLAG M2 affinity gel (Sigma–Aldrich) was added to the soluble fraction and incubated at 4 °C at constant rotation overnight. Then the affinity gel was washed with lysis buffer for four times. The immunoprecipitates were eluted with 100 μl of FLAG peptides and then denatured by boiling in SDS loading buffer for 10 min.

### SDS-PAGE Analysis and In-gel Protein Digestion

The immunoprecipitated samples were loaded onto SDS-PAGE, and electrophoresis was stopped shortly after the migration of protein samples into the resolving gel. Stained gels were processed into two or three gel slices per sample for further in-gel digestion, following the procedures described before (24). Briefly, gel slices were cut into 1 mm^3^ cubes and destained with 50% (v/v) acetonitrile (ACN) in 50 mM NH_4_HCO_3_. After dehydration by neat ACN, protein disulfide bonds were reduced with 10 mM DTT in 100 mM NH_4_HCO_3_ at 56 °C for 30 min and subsequently alkylated with 55 mM iodoacetamide in 100 mM NH_4_HCO_3_ at room temperature for 20 min (in complete darkness). In-gel protein digestion was carried out in a buffer containing 1.2 ng/μl trypsin and 10% (v/v) ACN in 50 mM NH_4_HCO_3_ for 16 h at 37 °C. The tryptic peptides were extracted from gel cubes twice by incubating with 50% (v/v) ACN and 5% (v/v) formic acid (FA) for 20 min at 37 °C. The resulting peptides were pooled and vacuum dried for further MS/MS analyses.

### LC–MS/MS Analyses of Peptide Samples

LC–MS analyses of peptide samples were carried out on a hybrid ion trap-Orbitrap mass spectrometer (LTQ-Orbitrap Velos; Thermo Scientific) coupled with nanoflow reversed-phase liquid chromatography (EASY-nLC 1200; Thermo Scientific). The capillary column (75 μm × 150 mm) with a laser-pulled electrospray tip (Model P-2000; Sutter Instruments) was home-packed with 5 μm, 120 Å Xtimate C18 silica-based particles (Welch) and run at 300 nl/min with the following mobile phases (A: 97% water, 3% ACN, and 0.1% FA; B: 80% ACN, 20% water, and 0.1% FA). The LC separation was carried out with the following gradient: solvent B was started at 7% for 3 min and then raised to 40% over 40 min; subsequently, solvent B was rapidly increased to 90% in 2 min and maintained for 10 min before 100% solvent A was used for column equilibration. Eluted peptides from the capillary column were electrosprayed directly onto the mass spectrometer for MS and MS/MS analyses in a data-dependent acquisition mode. One full MS scan (60,000 resolution, *m/z* 400–1200, 1 × 10^5^ automatic gain control target, and 500 ms maximal ion injection time) was acquired by the Orbitrap mass analyzer, and the 10 most intense ions were selected for fragmentation under collision-induced dissociation. Dynamic exclusion was set with repeat duration of 30 s and exclusion duration of 12 s.

### MS Data Processing

Label-free quantification (based on peptide ion intensity) was carried out by MaxQuant (version 1.5.4.1, Max-Planck-Institute of Biochemistry). MS/MS spectra were searched against the UniProt human protein database (Proteome IP UP000005640, version 2021_04) using the Andromeda search engine embedded in MaxQuant. The precursor mass tolerance was set at 20 ppm, and the fragment mass tolerance was set at 0.8 Da. The digestion enzyme was set as trypsin with a maximum of two missed cleavages. Cysteine carbamidomethylation (57.02 Da) was set as a fixed modification. Methionine oxidation (15.99 Da) was set as variable modifications. Both peptide and protein assignments were filtered to achieve a false discovery rate <1%. Only the proteins with at least two unique peptides were quantified. We removed protein hits that matched the reverse database as well as common contaminants. Logarithmic values (Log_2_) of label-free quantitation intensity were further processed using Perseus software (version 1.5.4.1, Max-Planck-Institute of Biochemistry), and the missing values (that refer to the scenario where a peptide signal is absent or not detected in one of the two paired samples) were replaced with random numbers from a normal distribution (width = 0.3 and shift = 1.8). The *p* values were obtained by using the two-tailed Student's *t* test. Proteins with *p* < 0.05 and average fold changes >2.0 were considered as hits with significant difference. Gene Ontology (GO) and Kyoto Encyclopedia of Genes and Genomes term enrichment were performed using clusterProfiler (R package), and terms with *p* values (hypergeometric test implemented in the package) <0.05 were considered as significant enrichment.

### Western Blot

The protein samples were separated by SDS-PAGE and transferred to polyvinylidene difluoride membranes. Immunoblotting analyses were carried out with primary antibodies specific for FLAG (1:2000 dilution; Cwbio), HA (1:2000 dilution; Cwbio), GFP (1:1000 dilution; Cwbio), γ-adaptin (1:1000 dilution; BD Biosciences), clathrin heavy chain (CHC; 1:10,000 dilution; Proteintech), ADP ribose (1:1000 dilution; Cell Signaling Technology), and horseradish peroxidase–conjugated secondary antibodies (1:5000 dilution; Cwbio), and the signals were obtained by Tanon-5200 Image System (Tanon). The band intensities were quantified using Fiji (25).

### Immunofluorescence

After indicated treatment, cells were fixed with 4% paraformaldehyde for 30 min at room temperature, washed with PBS, and permeabilized with 0.2% Triton X-100 in PBS for 15 min. Then, the cells were blocked with 5% goat serum for 1 h and stained with primary antibodies specific for trans-Golgi network glycoprotein 46 (TGN46) (1:200 dilution; Proteintech) in PBS containing 1% bovine serum albumin and 0.05% Tween-20 overnight at 4 °C. After stained with the secondary antibodies (1:500 dilution; Beyotime) for 1 h, the cells were washed with PBS, stained with 4′,6-diamidino-2-phenylindole for 5 min, and then washed three times with PBS. Slides were mounted with antifade mounting medium (Beyotime), and fluorescence images were taken on a NIKON ECLIPSE Ti microscope or Leica TCS SP8 multiphoton system.

The thermosensitive vesicular stomatitis virus G protein (VSVG) transportation assay was performed as described (26, 27). Briefly, HeLa cells were transfected with plasmids expressing EGFP-VSVG-ts045 and cultured at 40 °C for 20 h. Then the temperature was lowered to 32 °C. The cells were fixed at indicated time points (0, 0.5, 2, and 4 h) and then imaged by confocal fluorescence microscopy.

### Live-cell Imaging by Total Internal Reflection Fluorescence Microscopy and Spinning-disk Confocal Microscopy

The total internal reflection fluorescence (TIRF) microscope was based on a Nikon TIE microscope equipped with a CFI Apochromat TIRF 100× objective (1.49 numerical aperture; Nikon), a Perfect Focus Unit (Nikon), a Motorized XY stage (Prior Scientific), a fully enclosed and environmentally controlled cage incubator (Okolab), a motorized TIRF Illuminator Unit (Nikon), OBIS 488, 561, and 647 nm lasers (Coherent), the W-VIEW GEMINI Image splitting optics (Hamamatsu), a 1.5× tube lens, and an EMCCD camera (iXon Life 888; Andor Technology). The microscope was also equipped with a 100× oil objective (1.45 numerical aperture; Nikon), a CSU-X1 spinning disk confocal unit (Yokogawa), and an EMCCD camera (iXon Ultra 897; Andor Technology) on the left-side port. Imaging sequences were acquired using Micro-Manager 2.0 (28). The detection and tracking of clathrin-coated structures at the plasma membrane were accomplished by the cmeAnalysis software package (29). The tracks longer than 20 frames were used for the diffusion coefficient analysis using the MSDanalyzer package in MATLAB (MathWorks) (30).

### Transferrin Uptake

HeLa cells were incubated with 5 μg/ml of Alexa Fluor 568-conjugated transferrin (Thermo Fisher Scientific) at 37 °C for 7 min and then washed sequentially with ice-cold Dulbecco’s PBS and acid wash buffer (150 mM NaCl, 1 mM MgCl_2_, 0.125 mM CaCl_2_, and 0.1 M glycine, pH 2.5) for two times. Cells were then fixed with 4% paraformaldehyde at room temperature for 30 min, washed with Dulbecco’s PBS, and imaged by spinning-disk confocal microscopy (18 imaging planes spaced at 0.35 μm). The maximum z-projection of the first four planes near the bottom surface of the cell was created by Fiji. The mean fluorescence intensity of Alexa Fluor 568-conjugated transferrin in each cell expressing EGFP-vector or EGFP-SpvB was measured by Fiji.

### *S.* Typhimurium Infection

*S.* Typhimurium infection was performed when cell monolayers reached 70 to 85% confluence after siRNA knockdown. *S.* Typhimurium was grown overnight at 37 °C in LB medium with shaking. Bacterial cultures were diluted 1:20 in fresh LB medium and grown at 37 °C until an absorbance reached 0.9 at 600 nm. Bacteria were pelleted at 3000*g* and resuspended in Hank's balanced salt solution and were added to cells at a multiplicity of infection of 10. Infection was proceeded for 40 min at 37 °C in a 5% CO_2_ incubator. Cells were then washed twice with Hank's balanced salt solution and overlaid with DMEM containing 200 μg/ml gentamicin to kill extracellular bacteria. At 2 h after infection, the gentamicin concentration was reduced to 20 μg/ml.

For colony-forming units (CFUs) assays, at specific time points, cell monolayers were washed extensively with PBS and lysed in lysis buffer containing 20 mM Tris–HCl (pH 7.5), 150 mM NaCl, and 0.1% Triton X-100. Cell lysates was diluted with LB and plated on LB agar plates with streptomycin. CFU counting was performed 24 h later to enumerate intracellular bacteria.

For lactate dehydrogenase (LDH) release assays, cultured supernatants of HEK293T cells were collected at 18 h postinfection and measured with a commercial LDH kit according to the manufacturer’s protocols (Beyotime).

## Results

### SpvB Interacts with Clathrin and its Adaptor AP-1 *Via* its N-terminal Domain

To identify the host targets of SpvB, we expressed FLAG-SpvB in HEK293T cells and performed IP–MS analysis. Another *S*. Typhimurium T3SS effector, SopD2, was included as a control in this assay. Among >800 detected proteins, ∼200 proteins were preferentially enriched in the SpvB samples (fold changes >2, *p* <0.05). Kyoto Encyclopedia of Genes and Genomes pathway analysis highlighted cytoskeleton-associated proteins (*e.g.*, actin, dynamin) and TAB2, an adaptor required for c-Jun N-terminal kinase and NF-κB activation (Supplemental Fig. S1) (31). GO analysis revealed the enrichment of diverse biological processes and cellular functions (Supplemental Fig. S2).

**Fig. 1:**
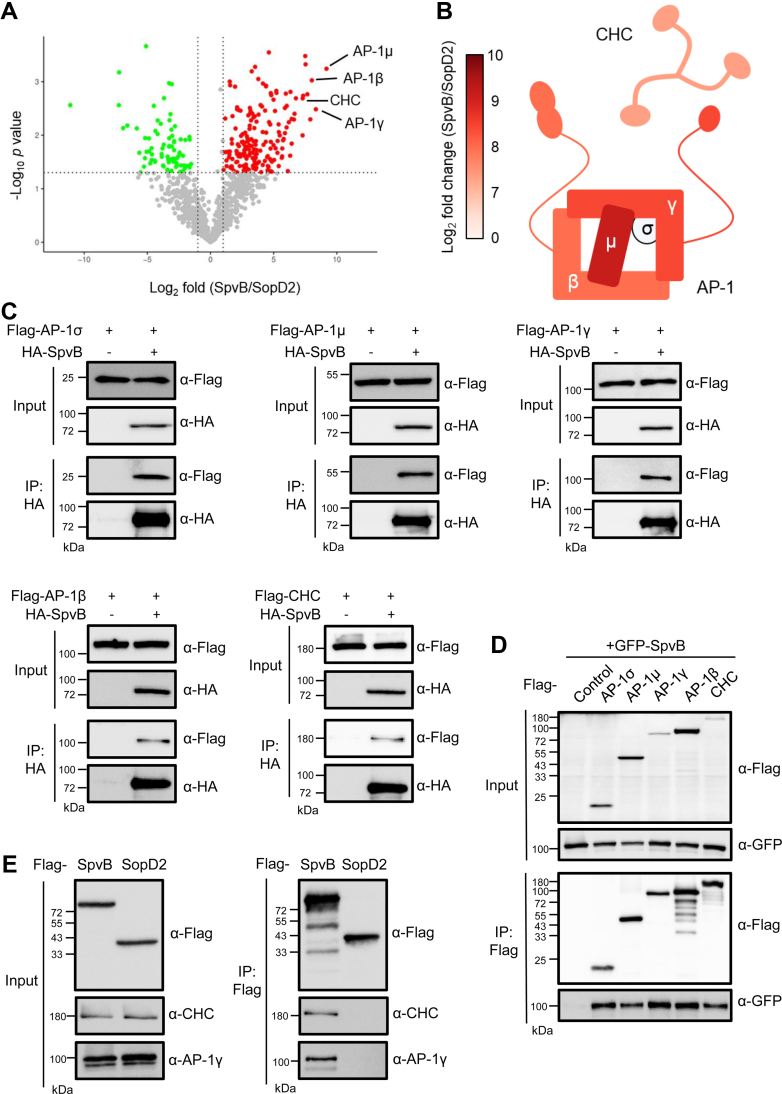
**Identification of CHC and AP-1 as SpvB-interacting proteins.***A*, a volcano plot of identified proteins in IP–MS analysis. Fold changes were calculated by dividing intensity of protein assignments from SpvB samples by those from SopD2 controls. The logarithmic values of fold changes and negative logarithmic values of *p* value were plotted on the *x*- and *y*-axis, respectively. Proteins with average fold changes >2 and *p* value < 0.05 were considered as significant hits. Candidate SpvB-binding substrates appear to the far right. *B*, *cartoon* summarizing IP–MS data (*i.e.*, CHC and AP-1) in (*A*). CHC and AP-1 subunits are color coded according to their fold changes. The *cartoon* was adapted from a figure in a review article (39). *C*, FLAG-tagged AP-1 or CHC was cotransfected with or without HA-SpvB in 293T cells. Lysates were immunoprecipitated with beads coated with HA antibody and immunoblotted with indicated antibodies. *D*, GFP-SpvB was cotransfected with or without FLAG-tagged AP-1 or CHC in 293T cells. Lysates were immunoprecipitated with beads coated with FLAG antibody and immunoblotted with indicated antibodies. *E*, 293T cells were transfected with FLAG-tagged SpvB or SopD2, and immunoprecipitated samples were probed with specific antibodies against endogenous CHC and AP-1γ. AP-1, adaptor protein 1; CHC, clathrin heavy chain; HA, hemagglutinin; IP–MS, immunoprecipitation–mass spectrometry.

Upon examining the volcano plot of all identified proteins (Fig. 1*A*), our attention was caught by a few distinct proteins (AP-1β, AP-1γ, and AP-1μ), which correspond to different subunits of the AP-1 complex. These AP-1 subunits showed abundant MS signals in immunoprecipitated SpvB samples but not SopD2 controls (Fig. 1, *A* and *B*), suggesting potentially specific interactions with SpvB. Functionally, the AP-1 complex is a clathrin adaptor and associated with clathrin-coated vesicles budding from the TGN and endosome (32). Intriguingly, strong MS signals of CHC were also measured in SpvB but not SopD2 IP samples. Consistently, clathrin coat–associated pathways were also enriched in GO analysis (Supplemental Fig. S2). To further investigate these interactions, we coexpressed HA-SpvB with FLAG-tagged AP-1 subunits or CHC in 293T cells. We included all four subunits (σ, μ, γ, and β) of AP-1 complex in co-IP experiments, though the MS signals of σ subunit were not abundant because of its small size. Indeed, immunoblotting analyses readily detected these individual AP-1 subunits as well as CHC in precipitates prepared by beads coated with HA antibody from SpvB-expressing cells but not controls (Fig. 1*C*). Consistently, reciprocal IP experiments showed that FLAG-tagged AP-1 subunits or CHC successfully pulled down GFP-SpvB but not controls (Fig. 1*D*). Finally, we verified the association of SpvB with endogenous host factors by probing the same sets of IP–MS samples with specific antibodies against CHC and one of the AP-1 subunits, AP-1γ. Consistently, immunoblotting data showed specific association of SpvB, but not SopD2, with CHC and its adaptor AP-1 complex (Fig. 1*E*). Taken together, these data suggest that the *S*. Typhimurium type III effector SpvB interacts with the vesicle coat protein clathrin as well as one of its adaptors, the AP-1 complex.

**Fig. 2:**
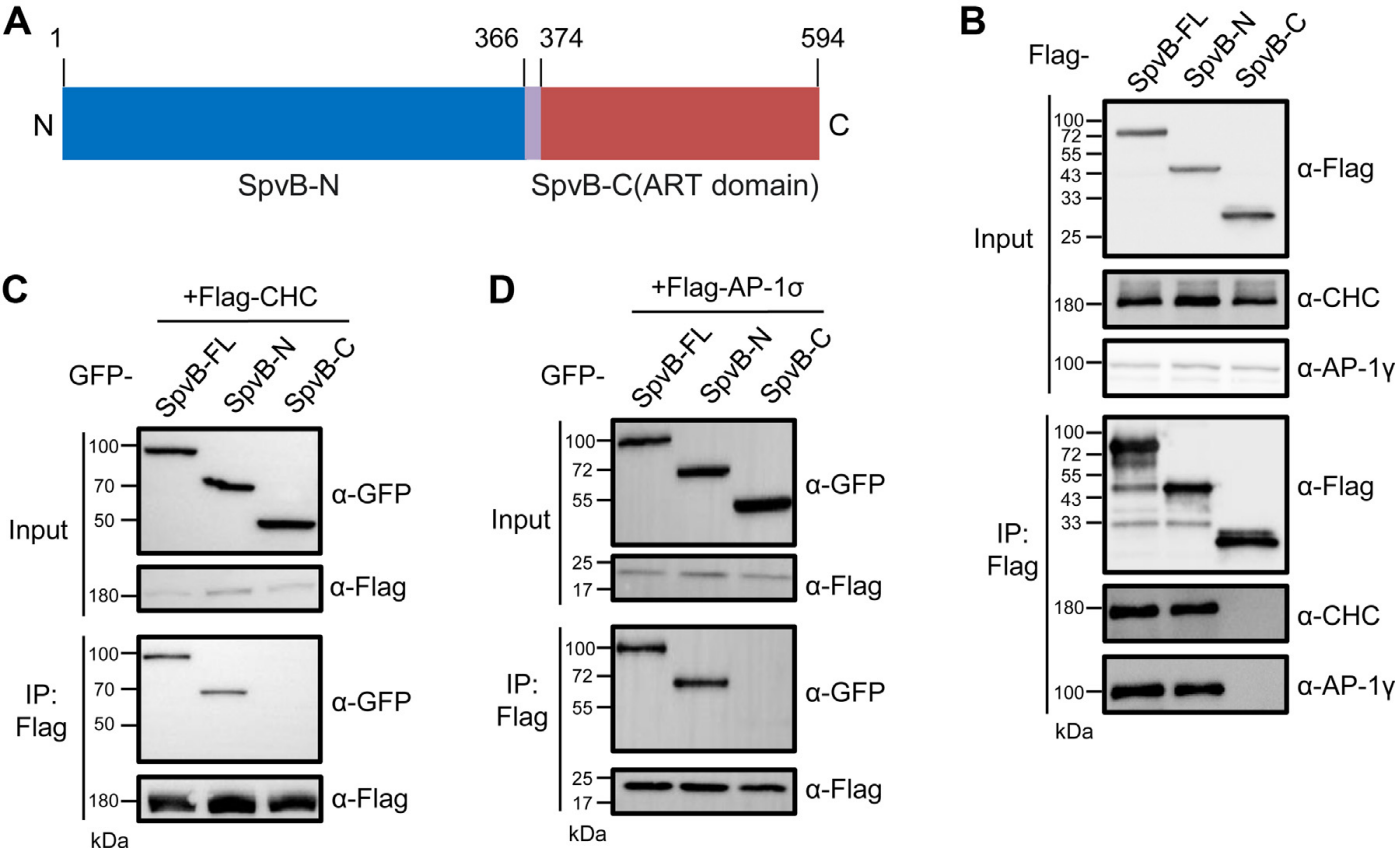
**CHC and AP-1 interact with the N-terminal domain of SpvB.***A*, schematic diagrams of SpvB truncation mutants used in this study. *B*, 293T cells were transfected with FLAG-tagged SpvB or truncation mutants, and immunoprecipitated samples were probed with antibodies against CHC and AP-1γ. *C* and *D*, GFP-tagged SpvB or truncation mutants were cotransfected with FLAG-tagged CHC (*C*) or AP-1σ (*D*) in 293T cells. Lysates were immunoprecipitated with beads coated with FLAG antibody and immunoblotted with indicated antibodies. AP-1, adaptor protein 1; CHC, clathrin heavy chain.

SpvB consists of a C-terminal ADP-ribosyltransferase domain and an N-terminal domain with unknown functions (Fig. 2*A*). Next we sought to determine which domain of SpvB is involved in the interactions we described previously. We generated two truncation mutants of SpvB expressing only the C-terminal or N-terminal domain and tested their interactions with endogenous CHC and AP-1γ. Immunoblotting assays showed that endogenous CHC and AP-1γ bind to the N-terminal domain as well as the full-length protein, whereas they fail to associate with the C-terminal enzymatic domain of SpvB (Fig. 2*B*). Likewise, reciprocal IP experiments revealed that FLAG-tagged CHC and AP-1σ readily pulled down the GFP-fused N-terminal domain or the full-length effector but not the C-terminal domain (Fig. 2, *C* and *D*). Collectively, these findings indicate that SpvB targets host clathrin and its adaptor protein AP-1 *via* the N-terminal domain.

### SpvB Does Not ADP-ribosylate Clathrin or AP-1 Complex

**Fig. 3:**
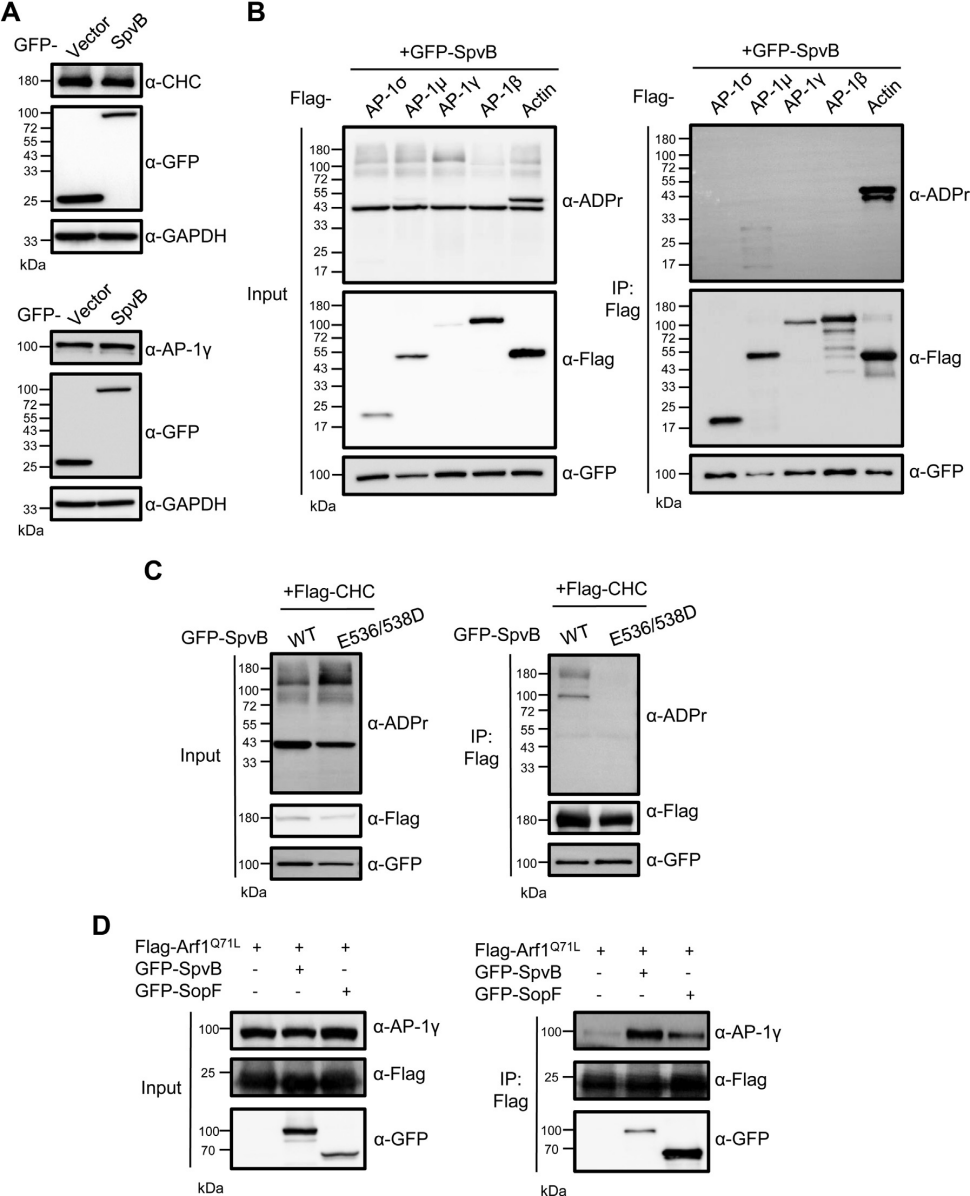
**SpvB does not ADP-ribosylate clathrin or AP-1 complex.***A*, 293T cells were transfected with GFP-tagged SpvB or vector, and cell lysates were probed with antibodies against CHC and AP-1γ. *B*, GFP-tagged SpvB was cotransfected with FLAG-tagged AP-1 subunits or actin in 293T cells. Lysates were immunoprecipitated with beads coated with FLAG antibody, and potential ADP-ribosylation was probed by an antibody specific against ADP-ribose. *C*, GFP-tagged SpvB or its catalytic mutant E536/538D was cotransfected with FLAG-tagged CHC in 293T cells. Lysates were immunoprecipitated with beads coated with FLAG antibody, and potential ADP-ribosylation was probed by an antibody specific against ADP-ribose. *D*, FLAG-tagged Arf1^Q71L^ was cotransfected with indicated effectors in 293T cells. Lysates were immunoprecipitated with beads coated with FLAG antibody and immunoblotted with indicated antibodies. AP-1, adaptor protein 1.

Next we asked whether the association of SpvB with CHC and AP-1 complex would affect their protein levels. Immunoblotting data revealed that coexpression of GFP-SpvB in mammalian cells did not detectably alter the abundance of clathrin or AP-1γ (Fig. 3*A*). As SpvB harbors the ADP-ribosyltransferase activity, we further sought to explore if SpvB can covalently modify these newly identified host targets. To test this hypothesis, we coexpressed GFP-SpvB with FLAG-tagged AP-1 subunits (AP-1σ, AP-1μ, AP-1γ, and AP-1β) in 293T cells. Actin was included as a positive control given that it is a known ADP-ribosylated substrate of SpvB. When lysates from SpvB-expressing cells were probed with an antibody specific for ADP-ribose, we were able to observe an intense band around 43 kDa, likely corresponding to ADP-ribosylated actin. The ectopically expressed actin was also modified as shown by a band slightly above the endogenous one. When immunoprecipitated substrates were further probed, we did not detectably observe any ADP-ribosylation signals of individual AP-1 subunits, though modified actin was readily measured (Fig. 3*B*). When FLAG-CHC coexpressed with GFP-SpvB was examined, immunoblotting analysis revealed a rather weak signal of ADP-ribosylation around 180 kDa in samples prepared from cells expressing the WT effector but not the catalytic mutant E536/538D (Fig. 3*C*). In light of the intense signals of ADP-ribosylated actin, these data would argue against clathrin being a physiologically relevant substrate of SpvB-mediated modification. Together, these results demonstrate that SpvB does not target AP-1 or CHC for ADP-ribosylation.

As a clathrin adaptor complex, AP-1 sorts cargo between TGN and endosomes, and its recruitment to these compartments depends on the small GTPase Arf1 *via* direct interaction (33). We next set out to determine whether SpvB affects the known AP-1–Arf1 interaction. We coexpressed the GTP-locked mutant Arf1^Q71L^ in 293T cells together with GFP-SpvB or SopF, a *S*. Typhimurium effector known to stably associate with Arf1 (34). Immunoprecipitated Arf1 samples were probed with an AP-1γ-specific antibody to assay the association of endogenous AP-1 complex. Immunoblotting data showed discernable yet more intense signals of AP-1γ in samples prepared from cells expressing SpvB than SopF or control cells without effector expression (Fig. 3*D*). These data indicate that SpvB, by interacting with AP-1 complex, may enhance its association with Arf1.

### SpvB Inhibits Clathrin-mediated Endocytosis and Secretory Pathways

**Fig. 4:**
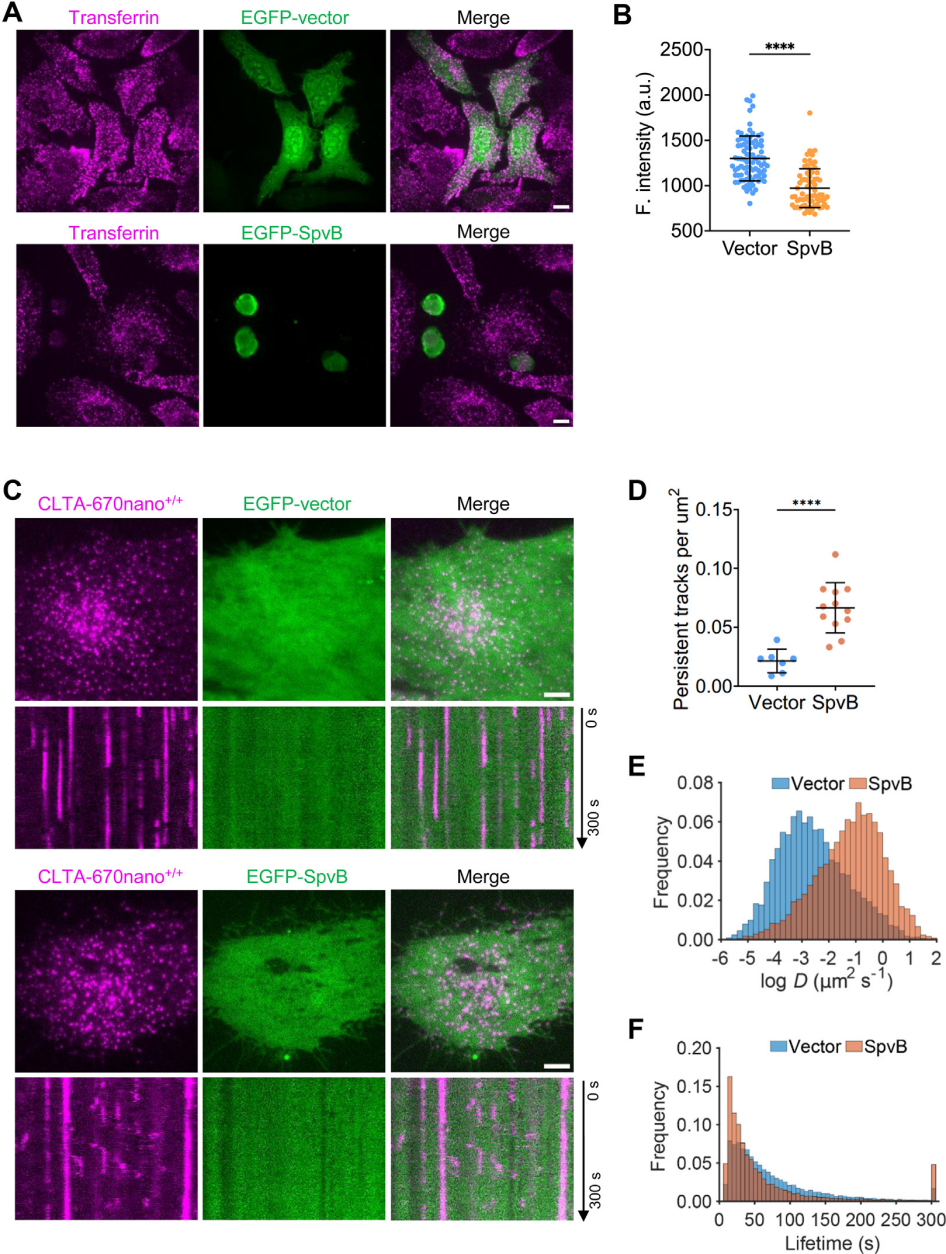
**SpvB disturbs the function and dynamics of clathrin-coated structures.***A*, representative images of the internalized Alexa Fluor 568-conjugated transferrin in the EGFP-vector or EGFP-SpvB expressing HeLa cells imaged by spinning-disk confocal microscopy (18 imaging planes spaced at 0.35 μm). The maximum z-projection of stacks are shown. Scale bars represent 10 μm. *B*, quantification of the fluorescence intensity of Alexa Fluor 568-conjugated transferrin in the EGFP-vector or EGFP-SpvB-expressing cells (mean ± SD; n = 87 and 70 cells). ∗∗∗∗*p* < 0.0001. *C*, SUM159 cells gene-edited for CLTA-670nano^+/+^ were transiently expressed with EGFP-vector (*top panels*) or EGFP-SpvB (*bottom panels*) and then imaged at the bottom surface by TIRF microscopy every 2 s for 300 s. A single frame and kymographs from a representative time series were shown. Scale bars represent 5 μm. *D*–*F*, the CLTA-670nano^+/+^ cells transiently expressing EGFP-vector or EGFP-SpvB were imaged as in (*C*) and then subjected to the automatic detection and tracking of clathrin-coated structures. The fraction of persistent tracks from seven EGFP-vector and 12 EGFP-SpvB-expressing cells (mean ± SEM; ∗∗∗∗*p* < 0.0001) (*D*), the distribution of the diffusion coefficient (*D*) of tracks (3025 and 3833 tracks from seven EGFP-vector and 12 EGFP-SpvB-expressing cells) (*E*) and the distribution of the lifetime of clathrin-coated structures (14,617 and 14,139 tracks from seven EGFP-vector and 12 EGFP-SpvB-expressing cells) (*F*) are shown. EGFP, enhanced GFP; TIRF, total internal reflection fluorescence.

Clathrin plays a key role in clathrin-mediated endocytosis (CME), which is the primary route for cargos to enter cells (22). Given the association of SpvB with CHC, next we sought to investigate whether such protein interactions would have any functional impact on CME by using the transferrin internalization assay. We monitored cellular uptake of fluorescently labeled transferrin into HeLa cells expressing enchanced green fluorescent protein-SpvB. Transferrin was readily internalized into the cells without SpvB expression or with control plasmid expression, whereas SpvB expression strongly inhibited the internalization of transferrin (Fig. 4, *A* and *B*).

**Fig. 5:**
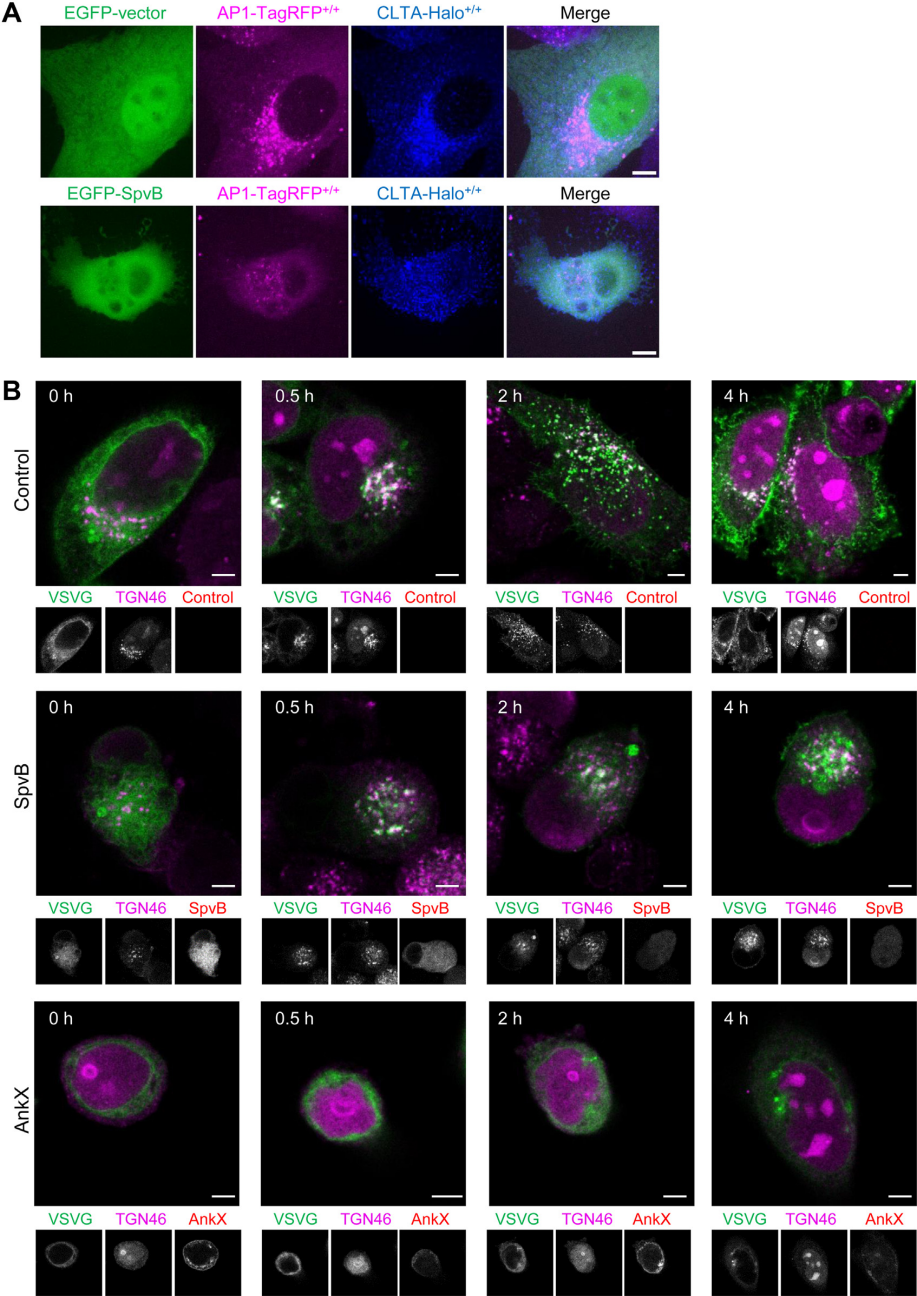
**SpvB disturbs the intracellular VSVG trafficking.***A*, SUM159 cells gene-edited for CLTA-Halo^+/+^ (labeled with the JFX_650_-HaloTag ligand) and AP1-TagRFP^+/+^ were transiently expressed with EGFP-vector (*top panels*) or EGFP-SpvB (*bottom panels*) and then imaged by spinning-disk confocal microscopy (18 imaging planes spaced at 0.35 μm). The maximum z-projection of stacks are shown. Scale bars represent 10 μm. *B*, representative images of VSVG-ts045 transport at different time points. HeLa cells cotransfected with EGFP-VSVG-ts045 and RFP-tagged SpvB or AnkX were cultured at 40 °C for 20 h. Then the temperature was lowered to 32 °C. At 0, 0.5, 2, and 4 h, cells were fixed, stained with the anti-TGN46 antibody, and then imaged by confocal microscopy. The EGFP-VSVG and TGN46 merged images are shown for each time points, with the gray image of each channel shown below. Scale bars represent 5 μm. EGFP, enhanced GFP; RFP, red fluorescent protein; VSVG, vesicular stomatitis virus G protein.

To gain more insight into how SpvB impacts host cell endocytosis, we examined the dynamics of clathrin-coated pits and vesicle formation in genome-edited SUM159 cells with clathrin light chain A fused to a fluorescent protein miRFP670nano (CLTA-670nano^+/+^) (35) by TIRF microscopy. By analyzing the dynamics of clathrin-coated structures at the plasma membrane of cells transiently expressing EGFP-SpvB or an empty vector, we found profound perturbation of the normal dynamics of clathrin-coated structures in SpvB-expressing cells (Fig. 4*C*). The fraction of clathrin-coated structures stayed persistently at the plasma membrane was significantly increased (Fig. 4*D*). Moreover, SpvB expression induced the generation of more short-lived structures with fast mobility (Fig. 4, *E* and *F*). We also examined the distribution of intracellular clathrin-coated structures formed on endosomes and TGN in the genome-edited cells expressing CLTA-Halo^+/+^ and AP-1-TagRFP^+/+^ by spinning-disk confocal microscopy. Interestingly, the distribution of intracellular clathrin- and AP1-coated structures around the perinuclear regions was perturbed by SpvB expression (Fig. 5*A*). Taken together, these results indicate that by binding and impairing the normal distribution and dynamics of clathrin, SpvB could negatively regulate the functions of clathrin-mediated membrane trafficking processes.

The clathrin/AP1-coated structures mediate the intracellular trafficking between the TGN and endosomes as well as from TGN to the plasma membrane (36). Next, we investigated the impact of SpvB on cargo export from the TGN using the EGFP-fused thermosensitive VSVG (VSVG-ts045) (27). As a viral glycoprotein, VSVG needs to be trimerized prior to its secretion. At the restrictive temperature (*i.e.*, 40 °C), the VSVG-ts045 mutant is unable to trimerize and thus retained in the endoplasmic reticulum. At the permissive temperature of 32 °C, it can traffic toward the plasma membrane through the TGN (27). HeLa cells were transfected to express red fluorescent protein-tagged SpvB or AnkX, a *Legionella* effector known to interfere with host vesicle trafficking (37). At 30 min upon temperature switch from 40 °C to 32 °C, VSVG was largely redistributed to the Golgi apparatus in both untransfected and SpvB-expressing cells but not in AnkX-expressing cells (Fig. 5*B*). In control cells, VSVG was further transported from TGN to plasma membrane (Fig. 5*B*). On the contrary, in the SpvB-expressing cells, the EGFP signal was still largely trapped in the TGN even at 4 h post-temperature shift (Fig. 5*B*). Collectively, these findings suggest that SpvB interferes with host protein secretory pathway by targeting the trafficking from the TGN to the plasma membrane.

### Host AP-1 Complex Restricts *S.* Typhimurium Intracellular Replication

**Fig. 6:**
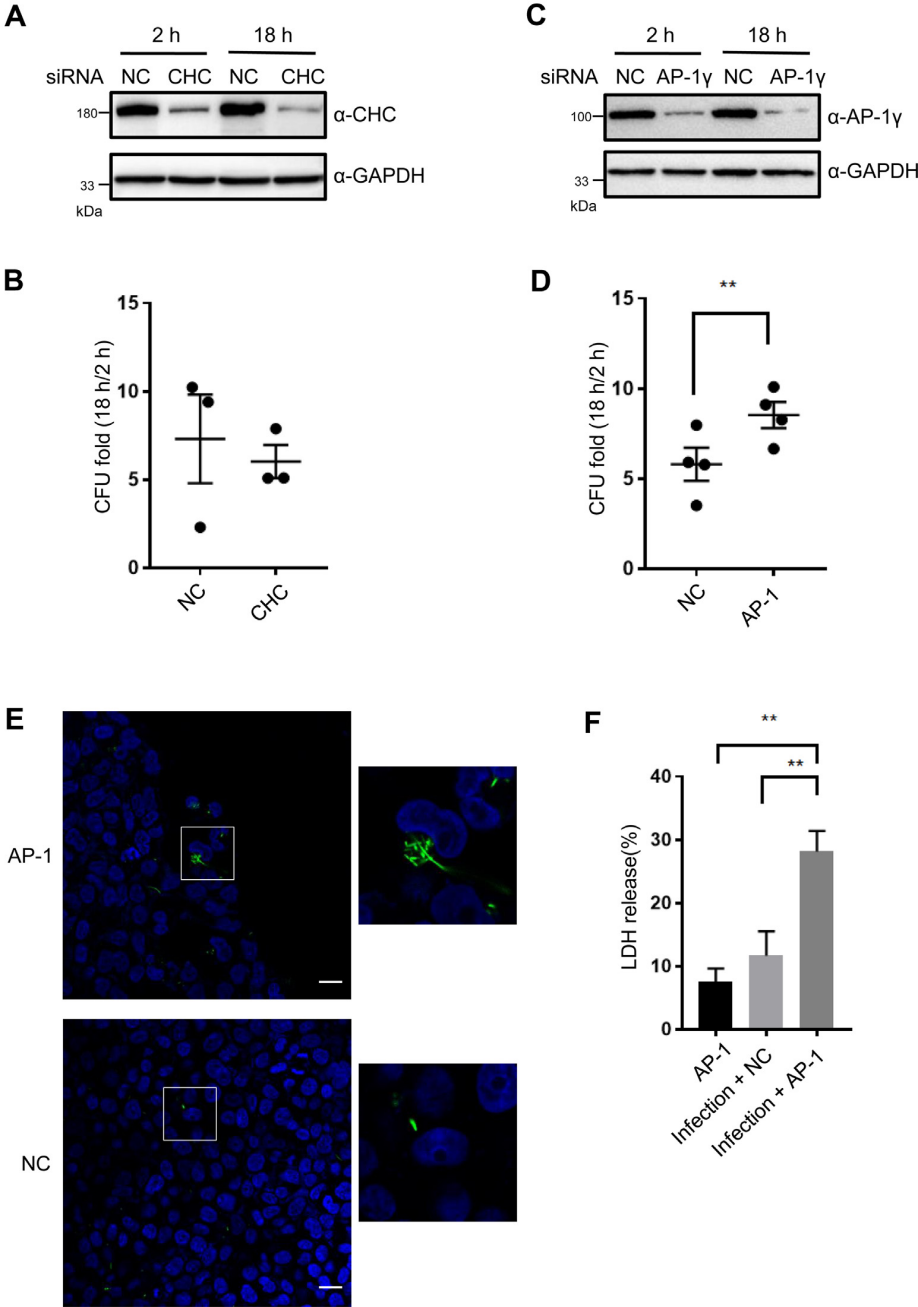
**AP-1 plays a role in host defense against *Salmonella* Typhimurium.***A* and *C*, immunoblotting analysis of CHC (*A*) and AP-1γ (*C*) in 293T cells transfected with siRNAs targeting CHC or AP-1γ at 2 h and 18 h post *S*. Typhimurium infection. HEK293T cells were transfected with siRNAs to deplete AP-1γ or CHC and 2 days later were infected with *S.* Typhimurium at MOI = 10. Cells transfected with nontargeting siRNAs were used as negative controls (NCs). Cell lysates were immunoblotted with antibodies against the CHC or AP-1γ 2 h or 18 h postinfection. *B* and *D*, *S.* Typhimurium replication in 293T cells depleted of CHC (*B*) or AP-1γ (*D*) by siRNAs. Fold of *S*. Typhimurium replication was evaluated by dividing bacterial CFUs at 18 h postinfection to those at 2 h postinfection. Results are the mean ± SEM of at least three independent determinations. ∗∗*p* < 0.01. *E*, representative images of EGFP-expressing *S.* Typhimurium within infected 293T cells treated with AP-1γ-targeting or nontargeting siRNAs at 18 h postinfection. *S.* Typhimurium was shown in *green*. Nuclei were stained with DAPI. Scale bars represent 20 μm. *F*, LDH release assays in 293T cells treated with AP-1γ-targeting or nontargeting siRNAs at 18 h postinfection. The assay was performed using a commercial LDH kit. Results are the mean ± SEM of at least three independent determinations. ∗∗*p* < 0.01. AP-1, adaptor protein 1; CFU, colony-forming unit; CHC, clathrin heavy chain; DAPI, 4′,6-diamidino-2-phenylindole; HEK293T, human embryonic kidney 293T cell line; LDH, lactate dehydrogenase; MOI, multiplicity of infection.

After establishing that SpvB impairs vesicular transport by targeting clathrin and its adaptor AP-1, next we examined the impact of these host factors on *S.* Typhimurium infection of host cells. We knocked down the expression of CHC or AP-1 in 293T cells by siRNA and challenged these cells with *S.* Typhimurium at a multiplicity of infection of 10. At 2 h or 18 h postinfection, we determined the number of intracellular *S.* Typhimurium by CFU assays. In cells treated with CHC-targeting siRNA, we found that *S.* Typhimurium grew at rates indistinguishable from those in nontargeting cells (Fig. 6, *A* and *B*), arguing against a role of clathrin in bacterial replication within host cells. In contrast, we found substantially increased replication of intracellular *S.* Typhimurium in AP-1γ-depleted cells (Fig. 6, *C* and *D*). Consistently, we observed significantly more signals of intracellular bacteria by fluorescence microscopy when AP-1γ-knockdown cells were infected with EGFP-expressing *S.* Typhimurium (Fig. 6*E*). In addition, by using LDH release assays, we found that more infected cells underwent cell death likely owing to higher bacterial load when AP-1γ was depleted (Fig. 6*F*). Taken together, these results demonstrate that the AP-1 complex may play a role in restricting *S.* Typhimurium intracellular proliferation in host cells.

## Discussion

The *S.* Typhimurium type III effector SpvB was originally identified as a mono-ADP-ribosyl transferase, which targets actin by ADP-ribosylation to manipulate the host cytoskeleton (13). Later, a number of studies reported its involvement in diverse host cellular pathways, including disruption of intestinal epithelial barrier integrity and dysregulation of iron metabolism (16, 18). However, other than actin, no additional cellular targets have been identified thereafter. In particular, the function of its N-terminal domain remains undetermined. Our results indicate that SpvB interacts with clathrin and its adaptor AP-1, though these host targets are not ADP-ribosylated by the effector. Notably, SpvB-CHC and SpvB–AP-1 interactions require the N-terminal domain but not the C-terminal domain of SpvB. The host cytoskeleton was not significantly altered in cells expressing the N-terminal domain (data not shown), suggesting that this domain does not target the same cellular pathway as the C-terminal domain. Consistently, our results show that the N terminus of SpvB acts as a scaffold to engage with CHC and AP-1.

Previous studies have shown that binding of cargo signal peptides to AP-1 induces a conformational change of its core domain, thereby promoting its interaction with GTP-Arf1 (38). In fact AP-1 specifically recognizes YXXØ and [DE]XXXL[LI] signals (39). As SpvB can enhance AP-1–Arf1 association, it is conceivable that SpvB may function in a similar mechanism. However, we were unable to test this hypothesis because mutation of the potential signal recognition sites of SpvB led to substantially reduced expression of the effector in 293T cells.

Host cells rely on vesicular trafficking systems for nutrient uptake and waste disposal. As a matter of fact, host membrane trafficking is frequently targeted by pathogens to facilitate their invasion and intracellular replication (40, 41). Clathrin-mediated vesicular transport selectively sorts cargos in multiple cell membrane structures such as the plasma membrane, TGN, and endosomes (42). CME mediates the uptake of surface receptors including nutrient, adhesion, and signaling receptors and their binding ligands. It also regulates the surface abundance and activity of transmembrane transporters (22). The abnormal dynamics of clathrin-coated structures at the plasma membrane and the impaired transferrin uptake in the SpvB-expressing cells suggest that CME is suppressed by SpvB. The inhibition of CME is likely to impact nutrition acquisition, leading to altered host metabolism as previously reported (16, 43).

Other bacterial pathogens target clathrin as well including *Listeria monocytogenes*, *Brucella abortus*, and *Staphylococcus aureus* (44, 45). Rather, clathrin is thought to promote bacterial invasion, although in a CME-independent mechanism given the bulky size of bacteria. That being said, clathrin-dependent pathways are not common routes for bacterial internalization. For example, *S.* Typhimurium injects T3SS effectors that engage with the host cytoskeleton to induce actin polymerization and membrane ruffling, eventually leading to bacterial internalization (46). Indeed, a previous report claimed that clathrin does not participate in the invasion of *S.* Typhimurium (44). Upon internalization into host cells, *S.* Typhimurium replicates in *Salmonella*-containing vacuoles (SCVs) (47). The formation of SCVs requires different sources of membrane components and nutrients. We found that knockdown of CHC did not adversely affect *S.* Typhimurium intracellular replication, suggesting that clathrin is unlikely to contribute to SCV maturation as well as maintaining their integrity.

As a clathrin adaptor, AP-1 mediates vesicular trafficking between the TGN and endosomes (48). Unlike the well-studied AP-2-mediated CME, however, AP-1-mediated intracellular transport has been less characterized. Recent studies have suggested that interference of AP-1 expression could potentially affect the trafficking of proteins to the plasma membrane in both the polarized and nonpolarized cells (49, 50). Consistent with this notion, our results suggest that SpvB severely impairs the export of cargos from the TGN to plasma membrane, but not the endoplasmic reticulum to Golgi transport, by a mechanism dependent on SpvB interactions with AP-1 or clathrin.

*S.* Typhimurium delivers the SPI-2 effector SteD to the major histocompatibility complex-II compartment by utilizing the AP-1-mediated vesicle transport pathway (51). Our immunofluorescence results show that SpvB is dispersed in host cells, indicating that SpvB–AP-1 interaction is unlikely to mediate the transport of SpvB itself. AP-1 is required for the correct localization of major histocompatibility complex-II in the plasma membrane (52). In addition, AP-1 is involved in the termination of STING-dependent immune signaling (34). Growing evidence suggests that AP-1-transported substrates are involved in restricting pathogen proliferation. Consistent with this notion, we found that knockdown of AP-1 facilitates the intracellular proliferation of *S.* Typhimurium. Therefore, it is conceivable that some molecules transported by AP-1 participate in the resistance to bacterial proliferation. Identification of these host antimicrobial factors would warrant further investigations in the future.

## Data Availability

The MS proteomics data have been deposited to the ProteomeXchange Consortium *via* the iProX partner repository with the dataset identifier PXD044057. The link to access the raw data: https://www.iprox.cn/page/project.html?id=IPX0006801000. For those protein assignments with a single unique peptide, the annotated spectra have been deposited to MS-Viewer on the ProteinProspector website with the following search key: srnrhbwy2h.

## Supplemental data

This article contains Supplemental data.

## Conflict of interest

The authors declare no competing interests.

## Supplemental Data

Supplemental Fig. S1 **Dot plot of Kyoto Encyclopedia of Genes and Genomes (KEGG) pathway analysis showing over-represented terms.** Colors denote *p* values, and dot sizes indicate counts. **Supplemental Fig. S2. Dot plot of Gene Ontology (GO) analysis showing over-represented GO terms.** Colors indicate *p* values, and dot sizes denote fold enrichment compared to the whole proteome. **Supplemental Fig. S3. Quantitative analyses of Western blot data in**Figure 1**.***A*, quantification of Flag band intensities in Fig. 1*C*. *B*, quantification of GFP band intensities in Fig. 1*D*. *Asterisks* mark bands with intensities statistically different from control groups. *C*, quantification of CHC and AP-1γ band intensities in Fig. 1*E*. Results are the mean ± S.E.M. of at least three independent determinations, ∗∗*p* < 0.01, ∗∗∗*p* < 0.001, ∗∗∗∗*p* < 0.0001. **Supplemental Fig. S4. Quantitative analyses of Western blot data in Figure 2.***A*, quantification of CHC and AP-1γ band intensities in Fig. 2*B*. *B*, quantification of GFP band intensities in Fig. 2*C*. *C*, quantification of GFP band intensities in Fig. 2*D*. Results are the mean ± S.E.M. of at least three independent determinations, ∗∗*p* < 0.01, ∗∗∗*p* < 0.001, ∗∗∗∗*p* < 0.0001. **Supplemental Fig. S5. Quantitative analyses of Western blot data in Figure 3.***A*, quantification of CHC and AP-1γ band intensities in Fig. 3*A*. *B*, quantification of ADP-r band intensities in Fig. 3*B*. *Asterisks* mark bands with intensities statistically different from the positive control actin. *C*, quantification of ADP-r band intensities in Fig. 3*C*. *D*, quantification of AP-1γ band intensities in Fig. 3*D*. Results are the mean ± S.E.M. of at least three independent determinations, ∗, *p* < 0.05, ∗∗, *p* < 0.01, ∗∗∗∗, *p* < 0.0001. **Supplemental Fig. S6. Quantitative analyses of Western blot data in Figure 6.***A*, quantification of CHC band intensities in Fig. 6*A*. *B*, quantification of AP-1γ band intensities in Fig. 6*C*. Results are the mean ± S.E.M. of at least three independent determinations, ∗∗*p* < 0.01.
