# Supplementary figures and images for: The Salmonella Typhimurium Effector SpvB Subverts Host Membrane Trafficking by Targeting Clathrin and AP-1
Source: Mol Cell Proteomics. 2023 Nov 2;22(12):100674. doi: 10.1016/j.mcpro.2023.100674 (PMC10696399; doi:10.1016/j.mcpro.2023.100674)

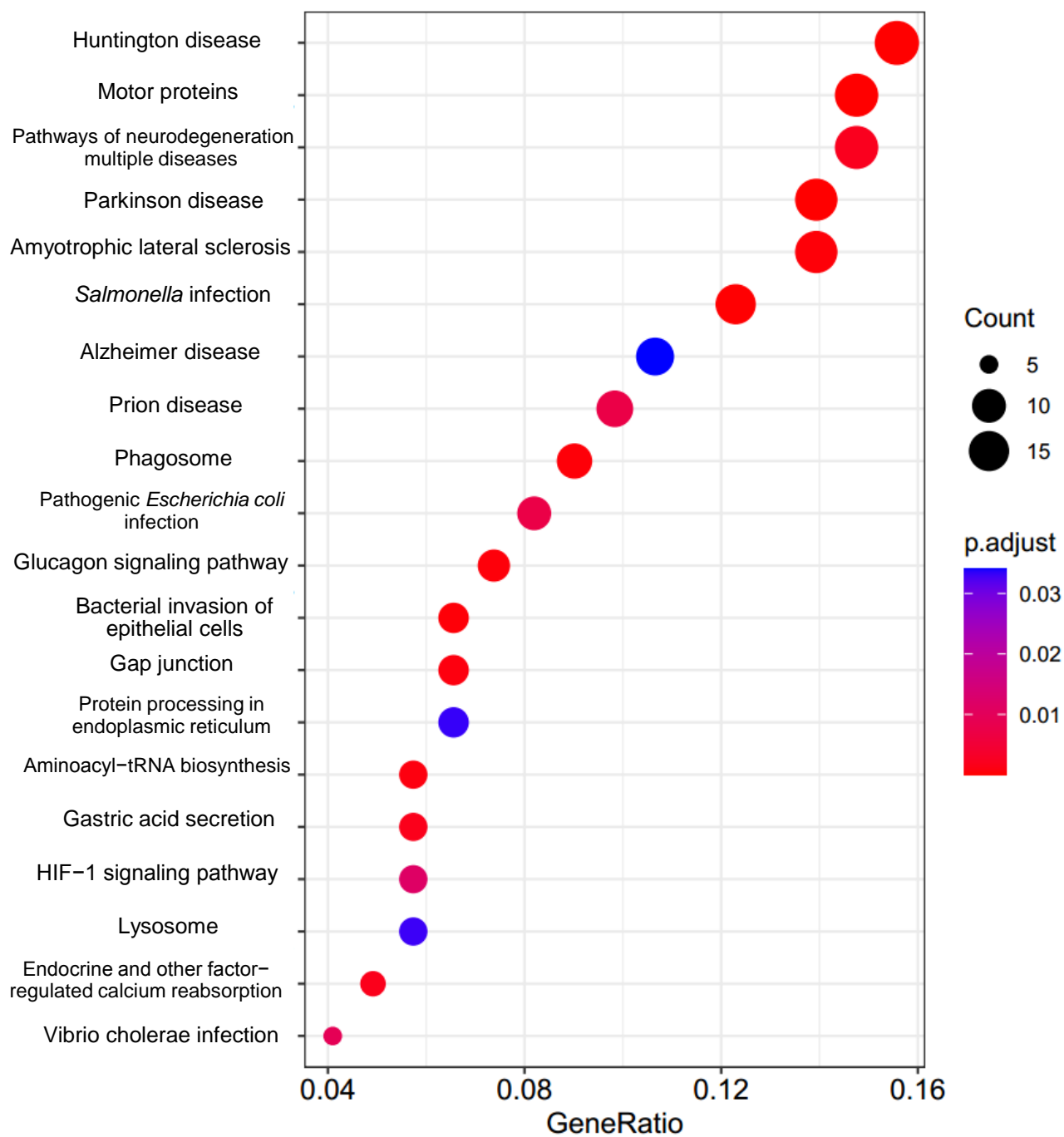

Fig. S1

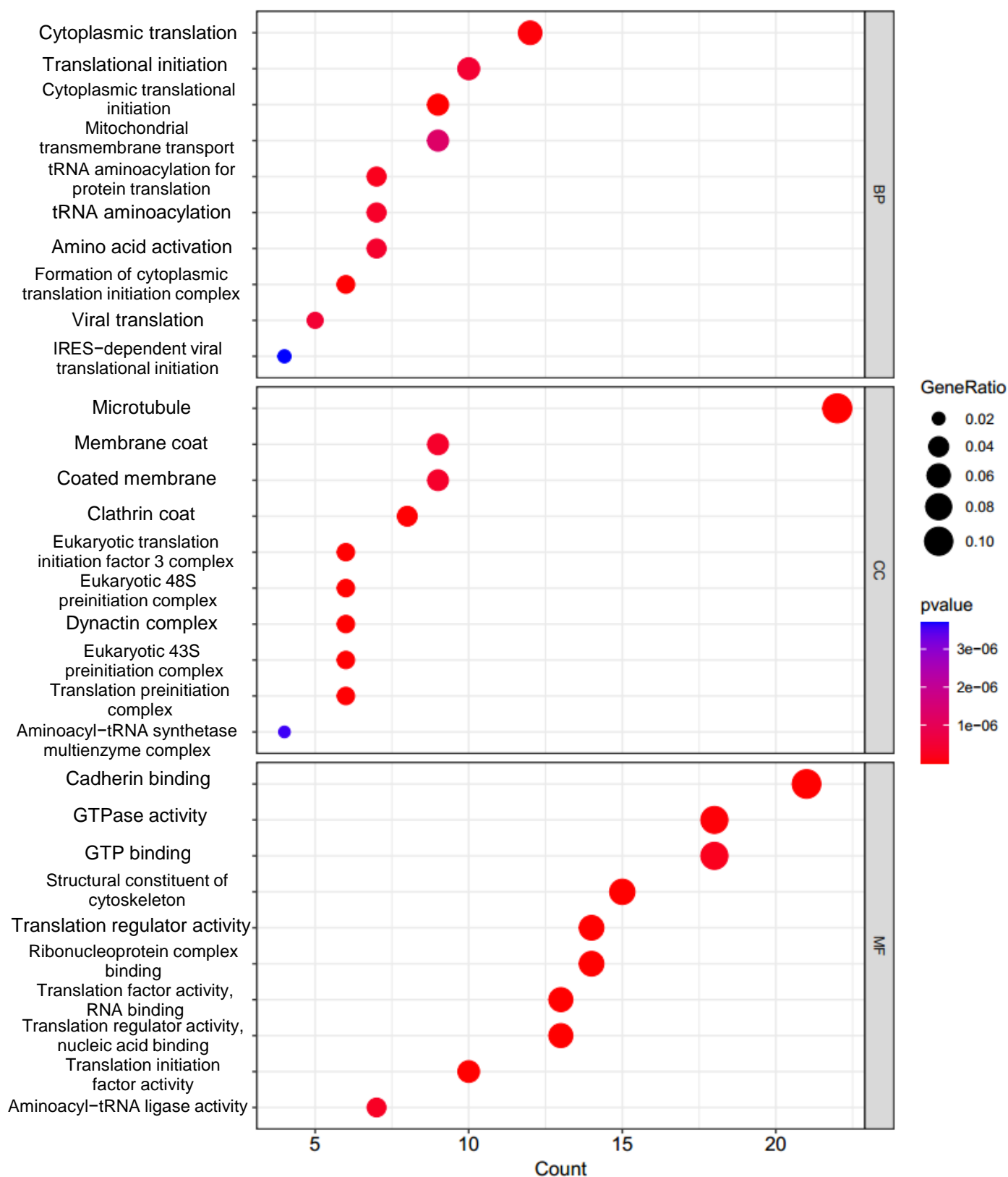

Fig. S2

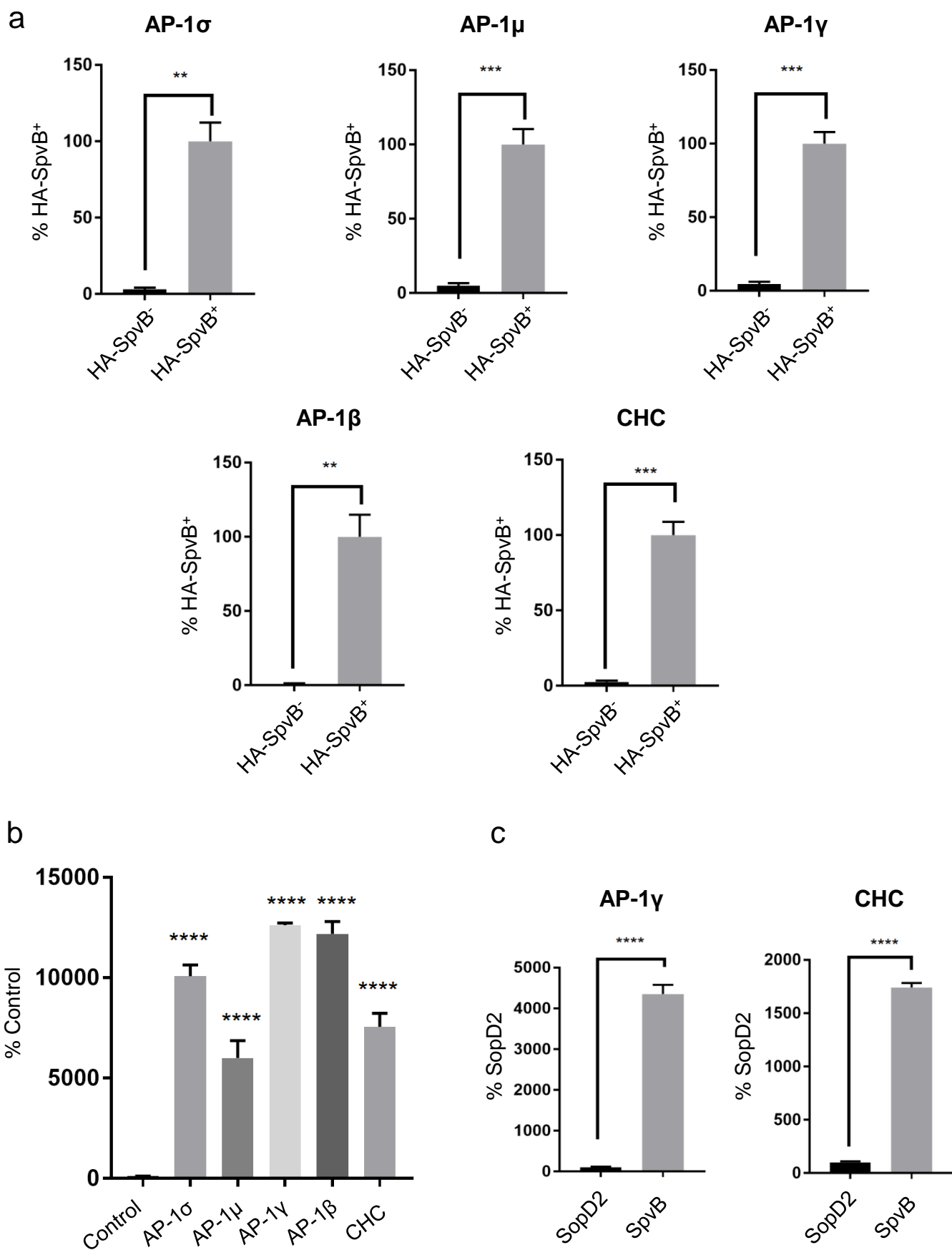

Fig. S3

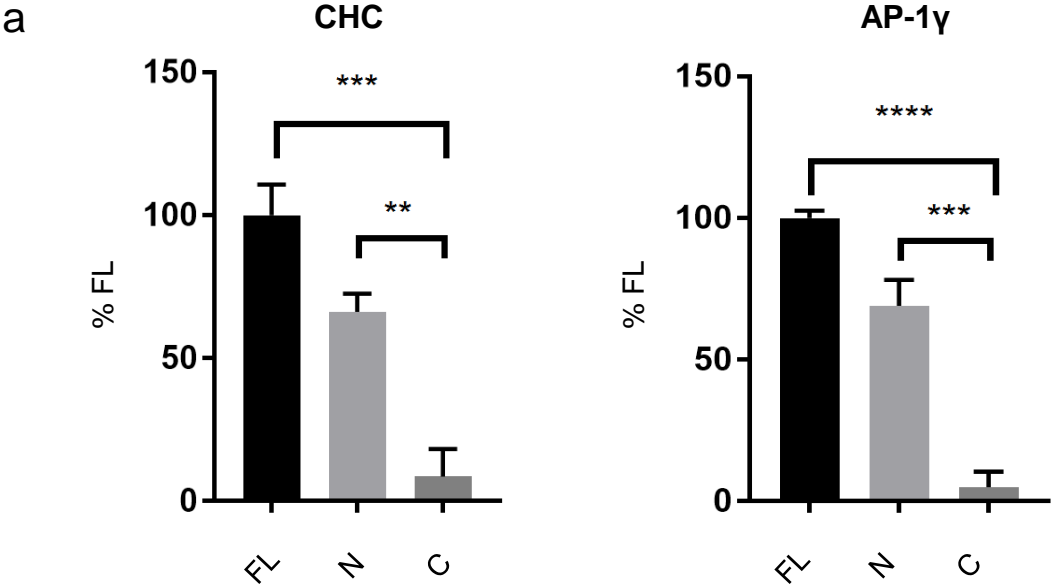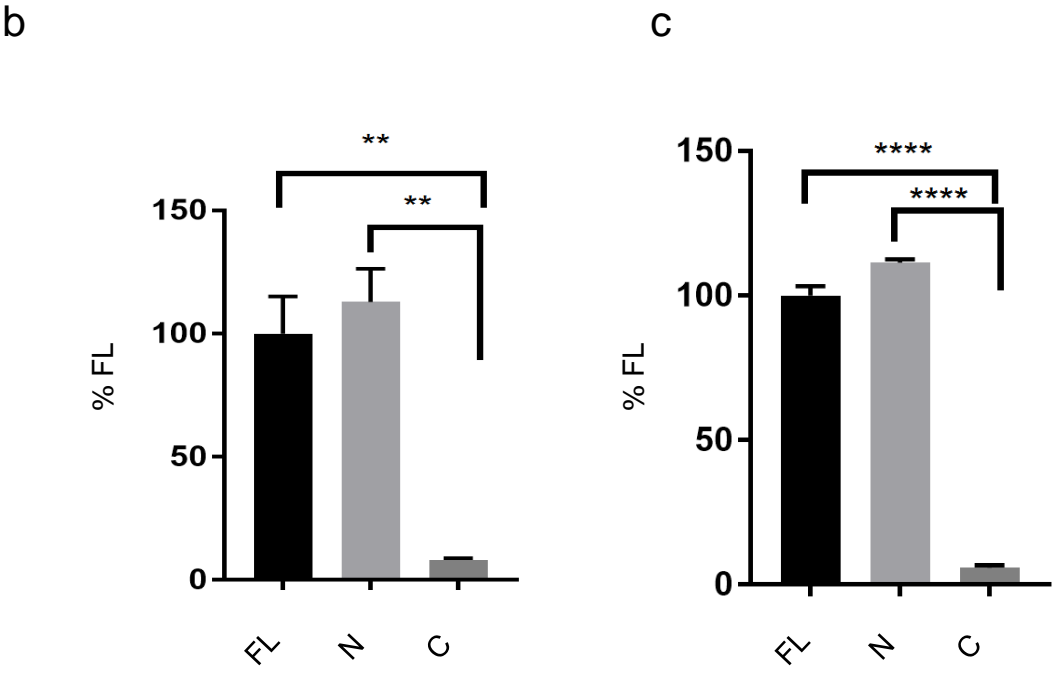

Fig. S4



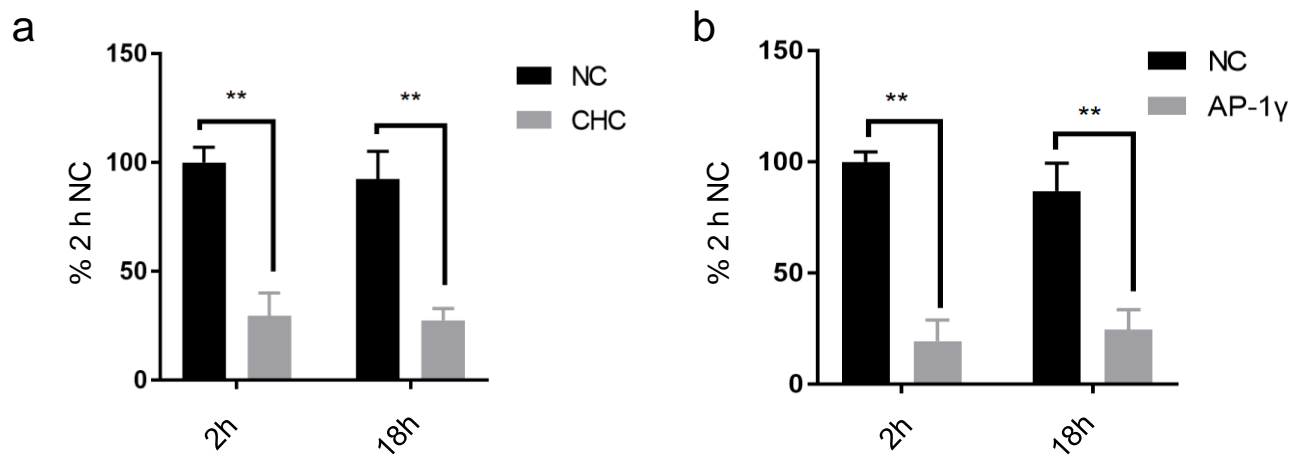

Fig. S6

Supplement: Supplemental Fig. S1 — Dot plot of Kyoto Encyclopedia of Genes and Genomes (KEGG) pathway analysis showing over-represented terms. Colors denote p values, and dot sizes indicate counts. Supplemental Fig. S2. Dot plot of Gene Ontology (GO) analysis showing over-represented GO terms. Colors indicate p values, and dot sizes denote fold enrichment compared to the whole proteome. Supplemental Fig. S3. Quantitative analyses of Western blot data inFigure 1.A, quantification of Flag band intensities in Fig. 1C. B, quantification of GFP band intensities in Fig. 1D. Asterisks mark bands with intensities statistically different from control groups. C, quantification of CHC and AP-1γ band intensities in Fig. 1E. Results are the mean ± S.E.M. of at least three independent determinations, ∗∗p < 0.01, ∗∗∗p < 0.001, ∗∗∗∗p < 0.0001. Supplemental Fig. S4. Quantitative analyses of Western blot data in Figure 2.A, quantification of CHC and AP-1γ band intensities in Fig. 2B. B, quantification of GFP band intensities in Fig. 2C. C, quantification of GFP band intensities in Fig. 2D. Results are the mean ± S.E.M. of at least three independent determinations, ∗∗p < 0.01, ∗∗∗p < 0.001, ∗∗∗∗p < 0.0001. Supplemental Fig. S5. Quantitative analyses of Western blot data in Figure 3.A, quantification of CHC and AP-1γ band intensities in Fig. 3A. B, quantification of ADP-r band intensities in Fig. 3B. Asterisks mark bands with intensities statistically different from the positive control actin. C, quantification of ADP-r band intensities in Fig. 3C. D, quantification of AP-1γ band intensities in Fig. 3D. Results are the mean ± S.E.M. of at least three independent determinations, ∗, p < 0.05, ∗∗, p < 0.01, ∗∗∗∗, p < 0.0001. Supplemental Fig. S6. Quantitative analyses of Western blot data in Figure 6.A, quantification of CHC band intensities in Fig. 6A. B, quantification of AP-1γ band intensities in Fig. 6C. Results are the mean ± S.E.M. of at least three independent determinations, ∗∗p < 0.01. [file mmc1.pdf]
